# The impact of direct oral anticoagulants on viscoelastic testing – A systematic review

**DOI:** 10.3389/fcvm.2022.991675

**Published:** 2022-11-07

**Authors:** Sebastian D. Sahli, Clara Castellucci, Tadzio R. Roche, Julian Rössler, Donat R. Spahn, Alexander Kaserer

**Affiliations:** ^1^Institute of Anesthesiology, University and University Hospital Zurich, Zurich, Switzerland; ^2^Department of Outcomes Research, Anesthesiology Institute, Cleveland Clinic, Cleveland, OH, United States

**Keywords:** DOAC, point-of-care, ROTEM, TEG, ClotPro, FXa inhibitor, FII inhibitor

## Abstract

**Background:**

In case of bleeding patients and in acute care, the assessment of residual direct oral anticoagulant (DOAC) activity is essential for evaluating the potential impact on hemostasis, especially when a timely decision on urgent surgery or intervention is required. Viscoelastic tests are crucial in a modern goal-directed coagulation management to assess patients’ coagulation status. However, the role of viscoelastic test to detect and quantify residual DOAC plasma levels is controversially discussed. The aim of this review was to systematically summarize the evidence of viscoelastic tests for the assessment of residual DOAC activity.

**Method:**

PubMed, Embase, Scopus, and the Cochrane Library were searched for original articles investigating the effect of rivaroxaban, apixaban, edoxaban, or dabigatran plasma levels on different viscoelastic tests of the adult population from database inception to December 31, 2021.

**Results:**

We included 53 studies from which 31 assessed rivaroxaban, 22 apixaban, six edoxaban, and 29 dabigatran. The performance of viscoelastic tests varied across DOACs and assays. DOAC specific assays are more sensitive than unspecific assays. The plasma concentration of rivaroxaban and dabigatran correlates strongly with the ROTEM EXTEM, ClotPro RVV-test or ECA-test clotting time (CT) and TEG 6s anti-factor Xa (AFXa) or direct thrombin inhibitor (DTI) channel reaction time (R). Results of clotting time (CT) and reaction time (R) within the normal range do not reliable exclude relevant residual DOAC plasma levels limiting the clinical utility of viscoelastic assays in this context.

**Conclusion:**

Viscoelastic test assays can provide fast and essential point-of-care information regarding DOAC activity, especially DOAC specific assays. The identification and quantification of residual DOAC plasma concentration with DOAC unspecific viscoelastic assays are not sensitive enough, compared to recommended anti-Xa activity laboratory measurements.

**Systematic review registration:**

[https://www.crd.york.ac.uk/prospero/display_record.php?RecordID=320629], identifier [CRD42022320629].

## Introduction

Direct oral anticoagulants (DOACs) are prescribed for stroke prevention in atrial fibrillation, for the prevention and treatment of venous thromboembolism and for secondary cardiovascular prevention (1, 2). Currently, five substances are approved with regional differences for clinical use by medical regulatory authorities: rivaroxaban, apixaban, edoxaban, and dabigatran (3). The prescription and use of DOACs is steadily increasing since the introduction in 2008 (4). They are taken orally as fixed-dose regimens with no regular monitoring required (5).

In acute care, the assessment of residual DOAC activity is essential for evaluating the potential impact on hemostasis, especially when a timely decision on urgent surgery or intervention is required (6–9). Residual DOAC plasma levels can be quantified by high-pressure liquid chromatography-tandem mass spectrometry (HPLC-MS) or by chromogenic anti-Xa and anti-IIa assays (10). However, both measurements are more time-consuming compared to point-of-care assays. HPLC-MS measurement requires on average 2 h, whereas specific DOAC anti-Xa assays deliver results within 30 min. No standardized point-of-care test is currently available to evaluate the anticoagulant effects of DOACs (7, 8). Viscoelastic tests are crucial in a modern-goal directed coagulation management to assess patients’ coagulation status (11, 12). The role of viscoelastic test to detect residual DOAC plasma levels in acute care is controversially discussed. Therefore, this systematic review compiles the existing evidence on the accuracy of point-of-care viscoelastic tests to assess residual DOAC effects.

### Viscoelastic assays

**FIGURE 1:**
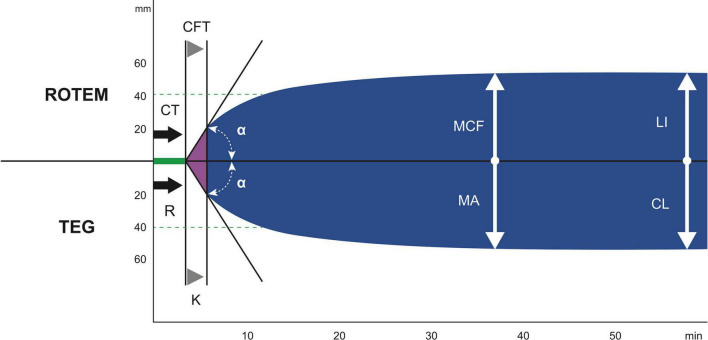
The difference in the terminology of the most important results between ROTEM and TEG. ROTEM parameters: CT, clotting time; CFT, clot formation time; α, α angle; MCF, maximum clot firmness; LI (30/60), lysis index 30 and 60 min after CT. TEG parameters: R, reaction time; k, kinetics; α, α angle; MA, maximum amplitude; CL (30/60), clot lysis after 30 and 60 min (89). With reprint permission by Georg Thieme Verlag KG.

Different from standard coagulation assays, viscoelastic tests are point-of-care systems analyzing in whole blood the process of clot formation and subsequent lysis in real time with on-line graphic display. The rotational thrombelastic system (ROTEM, Werfen, Bedford, MA, USA) and thrombelastographic system (TEG, Haemonetics Corporation, Boston, MA, USA) provide similar information but operate with different techniques. While ROTEM delta uses a rotating pin, the TEG 5000 uses a cup oscillating around the pin. The new ROTEM sigma operates by the same principle as delta but is automated with ready-to-use cartridges for simultaneous testing. The new TEG 6s system uses a resonance method, is fully automated and uses prefabricated cartridges, too. Differences in the terminology of the results between both devices are shown in Figure 1. ClotPro (Haemonetics Corporation, Boston MA, USA; formerly enicor GmbH, Munich, Germany) provides six channels for parallel testing. It has a unique Active-Tip technology with the dried reagents contained in a sponge at the pipette tip.

With the different viscoelastic systems, several assays can be performed depending on the clinical question (13). For ROTEM and ClotPro, the clotting time (CT) and for TEG the reaction time (R) is defined as the time from the beginning of the test until a clot firmness amplitude of 2 mm is achieved which reflects the velocity of thrombin generation.

## Methods

**FIGURE 2:**
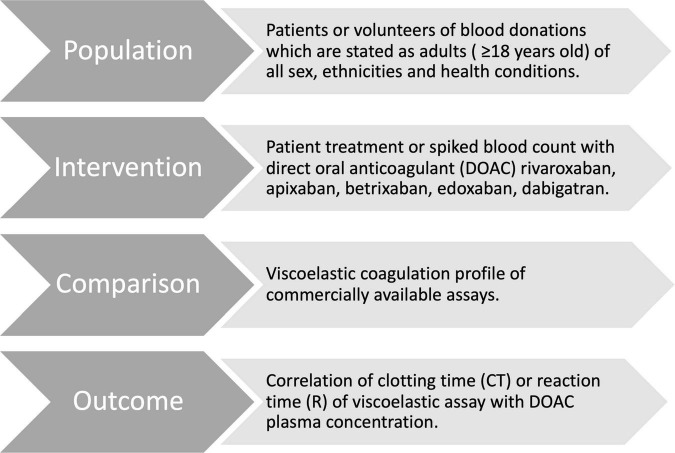
Clinical questions for evidence-based practice.

This systematic review follows the guidelines of PRISMA (Preferred Reporting Items for Systematic Reviews and Meta-Analyses) (Figure 2). We defined the PICO question for this review as “In adult patients taking DOACs (Population), are results in point-of-care viscoelastic tests (Intervention) compared to standardized laboratory tests or DOAC naïve blood (Control) altered by the drug (Outcome)?” This work was registered on the international prospective register of systematic reviews PROSPERO (registration ID # CRD42022320629).

### Eligibility criteria and study selection

We included original articles addressing the coagulation profile of DOACs assessed with viscoelastic assays of the adult population (>18 years old) from database inception to December 31, 2021. Articles were excluded if they did not consider apixaban, edoxaban, rivaroxaban, and dabigatran; did not involve whole blood viscoelastic assays; instruments for measuring activated clotting time (ACT) solely; or referred to animal studies. Non-commercially available assays were considered beyond the scope of this review and are mentioned, but not further described. Poster abstracts and case reports were excluded too.

### Search strategy

Four electronic databases have been queried: US National Library of Medicine (MEDLINE via PubMed), Excerpta Medica Database (EMBASE), Scopus database by Elsevier, and the Cochrane Library for Trials. We used following keywords and operators: (ROTEM or TEG or sonoclot or clotpro or reorox or viscoelastic or “viscoelastic hemostatic assay” or “viscoelastic test” or thrombelastometry or thrombelastography or thrombelastography or “hemostatic assay”) AND (DOAC or “direct oral anticoagulant” or NOAC or “new oral anticoagulants” or “non-vitamin k anticoagulants” or rivaroxaban or dabigatran or apixaban or edoxaban).

References of articles were retrieved for the inclusion of related articles. Publications in English and German language were considered.

### Selection process

Two reviewers (CC and SDS) examined studies independently by reading the titles and abstracts. The studies corresponding to the inclusion criteria were read and the reviewers abstracted data. Any discrepancies between the reviewers were resolved by discussion.

### Data items

A standard form was used to extract the following data: author(s), year of publication, study site and country, study design, number of overall patients, anticoagulant(s) examined, viscoelastic test(s) used, plasma concentrations of anticoagulant(s), main findings.

### Risk of bias

The methodological quality of each included article was evaluated by the Newcastle Ottawa Scale (NOS) (14) (Supplementary Table 1). Three independent authors performed the evaluation (SDS, CC, and TRR). Disagreement was solved by discussion.

### Statistics

We labeled the strength of the association, for absolute values of r, the following: 0 to 0.19 is regarded as very weak, 0.2 to 0.39 as weak, 0.40 to 0.59 as moderate, 0.6 to 0.79 as strong and 0.8 to 1 as very strong correlation (15). Regression analyses values of R^2^ were converted into an effect size f according to Cohen (16), where *f* = 0.10 represents a weak effect, *f* = 0.25 a moderate effect and *f* = 0.40 a strong effect (17).

## Results

**FIGURE 3:**
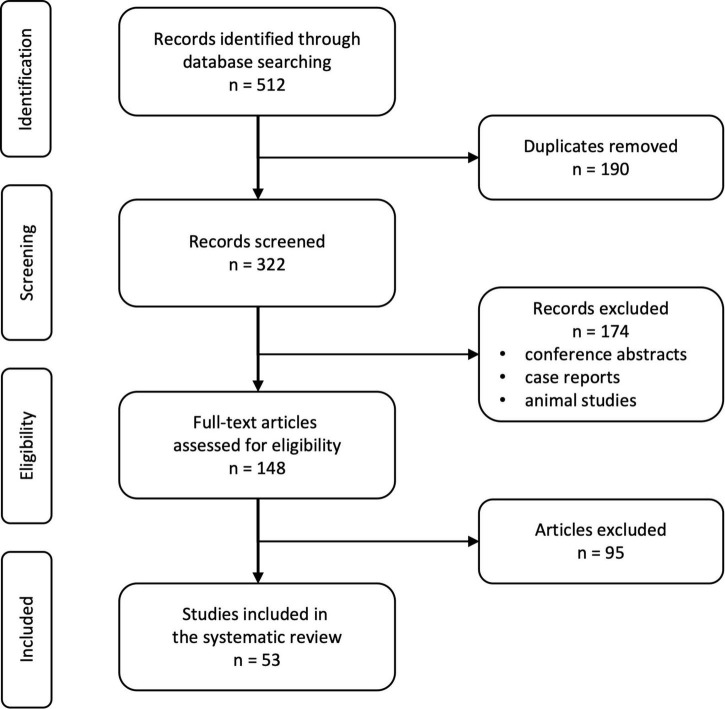
PRISMA flow chart of the selection process.

We identified 512 records in the mentioned databases of which 190 duplicates were automatically removed (Figure 3). The remaining 322 records were screened for suitability and a total of 148 full-text articles were proofread. A total of 53 studies met the pre-defined quality and inclusion criteria.

### Viscoelastic analysis of direct factor Xa inhibitors

#### Rivaroxaban

**FIGURE 4:**
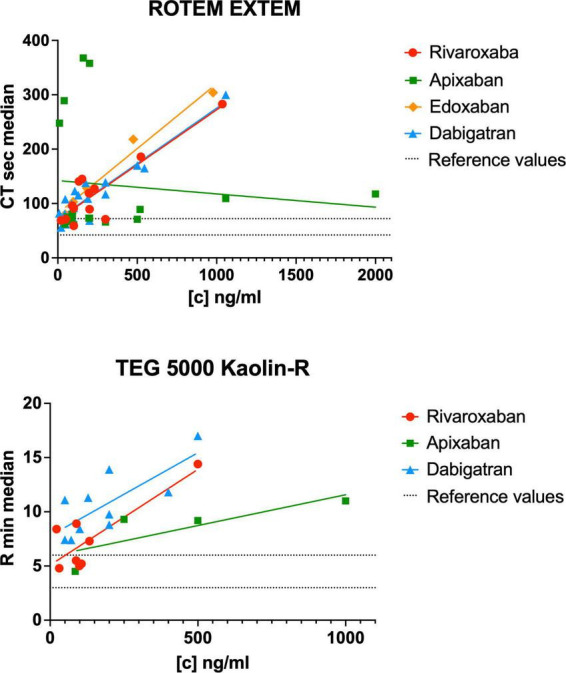
Reported clotting time (CT) and reaction time (R) in relation to the DOAC plasma concentration of the included studies for the ROTEM EXTEM and TEG 5000 kaolin assays. [c] DOAC plasma concentration in ng/ml. The dashed lines represent the lower and upper reference values for ROTEM EXTEM CT [42, 72 s (88)] and TEG 5000 R [3, 6 min (90)].

We identified 31 studies describing rivaroxaban action in whole blood with viscoelastic methods (18–48) (Supplementary Tables 2.1–2.3). Except for the well-documented viscoelastic parameters clotting time (CT) and reaction time (R), rivaroxaban showed either no significant influence or was not analyzed for other parameters besides individual and heterogenous nominations. The CT and R in relation to the rivaroxaban plasma concentration are shown in Figure 4.

##### Rivaroxaban and rotational thrombelastometry

Seventeen studies assessed rivaroxaban measurements in whole blood with ROTEM (Werfen, Bedford, MA, USA) (18, 19, 23, 24, 27, 28, 30, 31, 34, 36, 39–41, 44, 45, 47, 48) (Supplementary Table 2.1). A significant correlation between rivaroxaban plasma concentration and duration of clotting time (CT) was shown for EXTEM (strong to very strong correlation) (24, 28, 30, 34, 44, 45), INTEM (moderate to strong correlation) (24, 28, 30, 34, 45), NATEM (strong correlation) (19), FIBTEM (strong correlation) (44), and HEPTEM (strong correlation) (28) assays (Table 1.1). One study did not find any significant effect of rivaroxaban on ROTEM parameters (31).

**TABLE 1.1:** Study defined ROTEM clotting time (CT) thresholds for detection of the residual DOAC plasma concentration stratified by assays.

Assay	Threshold	CT	Sensitivity	Specificity	Study
	ng/ml	sec	%	%	
**Rivaroxaban**					
EXTEM	30	60	96	75	Henskens et al. (30)
	153	79	92	62	Chojnowski et al. (24)
INTEM	30	195	77	80	Henskens et al. (30)
	153	200	77	69	Chojnowski et al. (24)
NATEM	30	715	81	83	Aranda et al. (19)
*Modified*					
m-ROTEM	20	197 ^&^	85	100	Pailleret et al. (40)
	30	197 ^&^	90	85	Pailleret et al. (40)
	100	197 ^&^	96	64	Pailleret et al. (40)
LowTF	0	426	90	88	Adelmann et al. (18)
	200	524	98	96	Adelmann et al. (18)
**Apixaban**					
*Modified*					
m-ROTEM	20	197 ^&^	85	100	Pailleret et al. (40)
	30	197 ^&^	90	85	Pailleret et al. (40)
	100	197 ^&^	96	64	Pailleret et al. (40)
LowTF	0	432	96	97	Adelmann et al. (18)
	200	548	95	74	Adelmann et al. (18)
**Dabigatran**					
EXTEM	20	90	85	100	Taune et al. (69)
	30	60	91	75	Henskens et al. (30)
INTEM	30	195	52	50	Henskens et al. (30)
*Modified*					
Thrombin-b	20	154	100	100	Taune et al. (69)

^&^ Analysis pooled with apixaban and rivaroxaban cases. Reference ranges: 2.5 to 97.5 percentiles [median ] of EXTEM CT: 42 to 74 s [55 s]; INTEM CT: 137 to 246 s [184 s] (88). NATEM, non-activated rotational thromboelastometry; EXTEM, extrinsic activated rotational thromboelastometry; INTEM, intrinsic activated rotational thromboelastometry; Modified assays customized by study team; m-ROTEM, assay activated with tissue factor and phospholipid vesicles; LowTF, assay activated with low tissue factor; Thrombin-b, assay activated with thrombin-based trigger.

Three studies examined modified ROTEM assays (18, 40, 48). Some of these non-commercially available modified ROTEM activators showed promising results in detecting rivaroxaban plasma levels (Table 2.1).

**TABLE 1.2:** Study defined TEG 6s reaction time (R) thresholds for detection of residual DOAC plasma concentration stratified by assays.

Assay	Threshold	R	Sensitivity	Specificity	Study
	ng/ml	min	%	%	
**Rivaroxaban**					
AFXa channel	30	1.7	98	86	Artang et al. (21)
	50	2.1	95	80	Artang et al. (21)
	50	1.8	100	100	Artang et al. (20)
	50	1.95 ^&^	92	95	Bliden et al. (22)
	100	2.6	96	85	Artang et al. (21)
	100	3.4	100	91	Artang et al. (20)
**Apixaban**					
AFXa channel	30	2.5	100	100	Artang et al. (20)
	50	1.7	100	96	Artang et al. (21)
	50	2.5	100	92	Artang et al. (20)
	50	1.95 ^&^	92	95	Bliden et al. (22)
	100	2.6	100	63	Artang et al. (20)
	100	2.2	98	81	Artang et al. (21)
**Dabigatran**					
DTI channel	30	2.6	100	92	Artang et al. (21)
	30	2.1	92	100	Artang et al. (20)
	50	3.1	94	83	Artang et al. (21)
	50	2.5	100	90	Artang et al. (20)
	50	1.9	94	96	Bliden et al. (22)
	100	3.4	100	82	Artang et al. (21)
	100	3.0	100	96	Artang et al. (20)

^&^ Analysis pooled with apixaban and rivaroxaban cases. Normal reference range estimated using a non-parametric method (97.5% of population) and mean (SD) for AFXa R time: 0.6 to 1.5 min and 0.9 (0.2) min; DTI R time: 1.6 to 2.5 min and 2.0 (0.2) min (25). AFXa, anti-factor Xa channel; DTI, direct thrombin inhibitor channel.

**TABLE 1.3:** Study defined ClotPro clotting time (CT) thresholds for detection of residual DOAC plasma concentration stratified by assays.

Assay	Threshold	CT	Sensitivity	Specificity	Study
	ng/ml	sec	%	%	
**Rivaroxaban**					
RVV-test	50	177	90	100	Oberladstätter et al. (38)
	100	196	100	91	Oberladstätter et al. (38)
**Apixaban**					
RVV-test	50	136	80	88	Oberladstätter et al. (38)
	100	191	67	88	Oberladstätter et al. (38)
**Edoxaban**					
RVV-test	50	168	100	100	Oberladstätter et al. (38)
	100	188	100	75	Oberladstätter et al. (38)
**Dabigatran**					
ECA-test	50	189	100	90	Oberladstätter et al. (38)
	100	315	92	100	Oberladstätter et al. (38)

Reference range for CT in the ecarin test (ECA-test) is 68 to 112 sec, and that for the Russell ìs viper venom test (RVV-test) is 49 to 79 s (38). RVV-test, Russell’s viper venom activated test; ECA-test, ecarin activated test.

**TABLE 2.1:** Correlation of DOAC plasma concentration and ROTEM clotting time (CT) for different assays.

Assay	DOAC plasma concentration (ng/mL)	Coefficient	Study
**Rivaroxaban**			
EXTEM	0 to 1000	0.96	Seyve et al. (45)
	*NA*	0.83	Fontana et al. (28)
	84 (20 to 341); 206 (43 to 350)	0.69	Klages et al. (34)
	153 (107 to 198)	0.68	Chojnowski et al. (24)
	0 to 700	0.63	Schenk et al. (44)
	187 (± 139)	0.58	Henskens et al. (30)
INTEM	0 to 1000	0.86	Seyve et al. (45)
	187 (± 139)	0.69	Henskens et al. (30)
	*NA*	0.62	Fontana et al. (28)
	84 (20 to 341); 206 (43 to 350)	0.60	Klages et al. (34)
	153 (107 to 198)	0.56	Chojnowski et al. (24)
NATEM	18 (± 31); 185 (± 65)	0.79	Aranda et al. (19)
HEPTEM	*NA*	0.62	Fontana et al. (28)
FIBTEM	0 to 700	0.69	Schenk et al. (44)
*Modified*			
LowTF	60 to 420; 535 (± 147)	0.95	Adelmann et al. (18)
	60 to 420; 535 (± 147)	0.81	Adelmann et al. (18)
PiCT	60 to 420; 535 (± 147)	0.84	Adelmann et al. (18)
	60 to 420; 535 (± 147)	0.59	Adelmann et al. (18)
**Apixaban**			
EXTEM	0 to 1000	0.70	Seyve et al. (45)
INTEM	0 to 1000	0.77	Seyve et al. (45)
*Modified*			
LowTF	50 to 420; 64 (± 56)	0.96	Adelmann et al. (18)
	50 to 420; 64 (± 56)	0.81	Adelmann et al. (18)
PiCT	50 to 420; 64 (± 56)	0.60	Adelmann et al. (18)
	50 to 420; 64 (± 56)	0.38	Adelmann et al. et al. (18)
**Edoxaban**			
EXTEM	0 to 500	0.94	Havrdová et al. (55)
	0 to 1000	0.94	Seyve et al. (45)
INTEM	0 to 1000	0.92	Seyve et al. (45)
FIBTEM	0 to 500	0.91	Havrdová et al. (55)
**Dabigatran**			
EXTEM	0 to 1000	0.97	Seyve et al. (45)
	0 to 1000	0.95	Comuth et al. (58)
	74 (11 to 250); 120 (31 to 282)	0.92	Sokol et al. (64)
	86 (29 to 150); 175 (67 to 490)	0.92	Taune et al. (70)
	129 (81 to 204)	0.84	Herrmann et al. (31)
	34 (0 to 228); 82 (18 to 252)	0.77	Klages et al. (34)
INTEM	0 to 1000	0.93	Seyve et al. (45)
	0 to 1000	0.92	Comuth et al. (58)
	107 (91 to 305)	0.88	Körber et al. (60)
	74 (11 to 250); 120 (31 to 282)	0.84	Sokol et al. (64)
	34 (0 to 228); 82 (18 to 252)	0.79	Klages et al. (34)
	87 (29 to 150); 175 (67 to 490)	0.72	Taune et al. (69)
	129 (81 to 204)	0.68	Herrmann et al. (31)
FIBTEM	0 to 1000	0.98	Comuth et al. (58)
	88 (29 to 150); 175 (67 to 490)	0.93	Taune et al. (69)
*Modified*			
ECATEM	107 (91 to 305)	0.90	Körber et al. (60)
	47 (28 to 147)	0.77	Körber et al. (60)
	9 (0 to 59)	0.65	Körber et al. (60)
LowTF	89 (29 to 150); 175 (67 to 490)	0.36	Taune et al. (70)

DOAC plasma concentration presents as median and interquartile range, mean and standard deviation, or total range. Coefficients refer to correlation test of the original study either to Spearman’s, Pearson’s, or regression analysis. NATEM, non-activated rotational thromboelastometry; EXTEM, extrinsic activated rotational thromboelastometry; INTEM intrinsic activated rotational thromboelastometry; HEPTEM, intrinsic activated rotational thromboelastometry with added heparinase; FIBTEM, extrinsic activated rotational thromboelastometry with added cytochalasin D; Modified assays customized by study team; LowTF, assay activated with low tissue factor; PiCT, prothrombinase induced clotting time reagent; ECATEM, uses ecarin to initiate rotational thromboelastometry.

##### Rivaroxaban and thrombelastography

Thirteen studies described rivaroxaban action in whole blood with TEG (Haemonetics Corporation, Boston MA, U.S.A.) (20–22, 25, 26, 31–33, 35, 37, 42, 43, 46) (Supplementary Table 2.2). Of these, four studies did not report the exact rivaroxaban concentration under which conditions the viscoelastic tests were performed (22, 25, 32, 33). Four studies investigated with the new cartridge-based anti-factor-Xa [AFXa] channel of TEG 6s (20–22, 25), to the best of our knowledge not yet commercially available at the time of manuscript preparation (Table 1.2).

A significant correlation between rivaroxaban plasma concentration and reaction time (R) have been reported for the specific anti-factor-Xa channel [AFXa] (strong to very strong correlation) (20, 21, 37), and for citrated kaolin channel (moderate to strong correlation) (35, 37).

The study of Kaaber et al. mentions that approximately 65% of the patients treated with rivaroxaban presented with RapidTEG*™* activated clotting time within the normal reference, but that those with an activated clotting time above this level had a significantly increased risk of severe bleeding with high transfusion demands (33). Three studies did not find any significant effect of rivaroxaban on TEG 5000 parameters (31, 32, 42). One study compared the results descriptively only (46) (Table 2.2).

**TABLE 2.2:** Correlation of DOAC plasma concentration and TEG reaction time (R) for different assays.

Assay	DOAC plasma concentration (ng/mL)	Coefficient	Study
**Rivaroxaban**			
AFXa channel	206 (94 to 318)	0.93	Artang et al. (20)
	29 to 99	0.92	Artang et al. (21)
	88 (27 to 221)	0.68	Myers et al. (37)
Kaolin-TEG	88 (27 to 221)	0.67	Myers et al. (37)
	99 (48 to 311)	0.54	Kopytek et al. (35)
**Apixaban**			
AFXa channel	29 to 99	0.84	Artang et al. (21)
	104 (74 to 145)	0.83	Artang et al. (20)
Kaolin-TEG	85 (40 to 105)	0.55	Kopytek et al. (35)
**Dabigatran**			
DTI channel	92 (41 to 197)	0.94	Artang et al. (20)
	29 to 99	0.93	Artang et al. (21)
Kaolin-TEG	0 to 400	0.89	Solbeck et al. (67)
	71 (39 to 98)	0.79	Kopytek et al. (35)
	269 (54 to 837), 179 (26 to 687)	0.74	Solbeck et al. (65)
CaCl_2_ TEG	90 (± 71)	0.54	Pipilis et al. (63)

DOAC plasma concentration presents as median and interquartile range, mean and standard deviation, or total range. Coefficients refer to correlation test of the original study either to Spearman’s, Pearson’s, or regression analysis. AFXa, anti-factor Xa channel; DTI, direct thrombin inhibitor channel; Kaolin-TEG, intrinsic activated assay; CaCl_2_ TEG, contains calcium chloride solution.

##### Rivaroxaban and ClotPro analyzer

Two studies reported results from the ClotPro analyzer (29, 38) (Supplementary Table 2.3). The commercially available Russel’s viper venom test (RVV-test) for the detection of factor Xa antagonists showed strong to very strong correlations between rivaroxaban plasma concentration and clotting time (CT) (29, 38) (Tables 1.3, 2.3).

##### Viscoelastic thresholds for rivaroxaban concentrations

**FIGURE 5:**
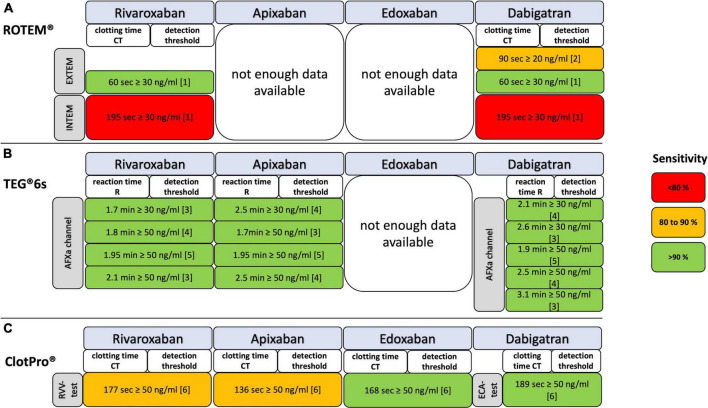
Detection of residual DOAC plasma concentrations with ROTEM, TEG, and ClotPro assays coded for test accuracy by sensitivity. **(A)** EXTEM, ROTEM assay extrinsic activated; INTEM, ROTEM assay intrinsic activated; NATEM, ROTEM assay natively activated; **(B)** DTI, direct thrombin inhibitor channel of TEG 6s; AFXa, anti-factor-Xa channel of TEG 6s; **(C)** ECA, ecarin-activated ClotPro test; RVV, Russell’s viper venom-activated ClotPro test. References: [1] Henskens et al. (30); [2] Taune et al. (69); [3] Artang et al. (21); [4] Artang et al. (20); [5] Bliden et al. (22); [6] Oberladstätter et al. (38).

Rivaroxaban plasma concentrations between 30 and 150 ng/ml can be detected by threshold values of the viscoelastic parameters CT and R (18–22, 24, 30, 38, 40) (Figure 5 and Tables 1.1–1.3). In particular, the RVV-test of ClotPro and the AFXa channel of TEG 6s show high sensitivity and specificity. It is important to mention that the study of Bliden et al. analyzed the results of rivaroxaban and apixaban in a pooled setting (22).

#### Apixaban

Twenty-two studies were identified analyzing the effects if apixaban on viscoelastic testing (18, 20–22, 25–27, 29, 32, 33, 35, 38, 40, 45, 46, 48–54) (Supplementary Tables 2.1–2.3). The CT and R in relation to the apixaban plasma concentration are shown in Figure 4.

##### Apixaban and rotational thrombelastometry

We revealed nine studies assessing the effects of apixaban on ROTEM (18, 27, 40, 45, 48–52) (Supplementary Table 2.1). Out of these, three studies used modified or *ad hoc* designed assays (18, 40, 48), whereas the other studies focused on the EXTEM (27, 45, 49–52), INTEM (27, 45), NATEM (50) and FIBTEM (45) assays. Apixaban caused a statistically significant prolongation of CT in EXTEM (27, 49, 51), with a greater sensitivity than CT in INTEM (27, 45). In comparison to the other factor Xa inhibitors, apixaban had the lowest effect on CT, often requiring supra-therapeutic doses to achieve a significant CT prolongation (27, 45). Also, apixaban plasma levels beneath 50 ng/ml could not be detected by EXTEM CT changes (45, 51). The significance of a dose dependent effect of apixaban plasma concentrations on CT length was shown for EXTEM [strong correlation (45)], INTEM [strong correlation (45)], and NATEM [weak correlation (50)] assays (Table 2.1).

Our search identified four studies analyzing modified ROTEM assays (18, 40, 48, 50). Overall, the experimental changes to ROTEM resulted in CT prolongation, with some studies requiring lower concentrations than those done with commercially available channels (Table 1.1).

##### Apixaban and thrombelastography

Ten studies investigated the effects of apixaban on TEG with a focus on R value, using either the TEG 6s anti-factor Xa channel [AFXa] (20–22, 25), or TEG 5000 system (26, 32, 33, 35, 46, 53, 54) (Supplementary Table 2.2). A statistically significant, dose-dependent correlation of reaction time (R) and apixaban plasma concentration was shown with the anti-factor Xa channel [very strong correlation (20, 21)] and kaolin-TEG [moderate correlation (35)] (Tables 1.2, 2.2).

Two studies reported no statistically significant effect of apixaban plasma levels on reaction time (R) (32, 53).

##### Apixaban and ClotPro analyzer

Using the ClotPro analyzer, Oberladstätter et al. (38) found a statistically strong correlation between apixaban plasma concentrations and clotting time (CT), which, compared to the other anti-factor Xa inhibitors used in the study, showed weaker correlation (Tables 1.3, 2.3, and Supplementary Table 2.3).

**TABLE 2.3:** Correlation of DOAC plasma concentration and ClotPro clotting time (CT) for different assays.

Assay	DOAC plasma concentration (ng/ml)	Coefficient	Study
**Rivaroxaban**			
RVV-test	0 to 650	0.88	Oberladstätter et al. (38)
**Apixaban**			
RVV-test	0 to 400	0.74	Oberladstätter et al. (38)
**Edoxaban**			
RVV-test	0 to 450	0.93	Oberladstätter et al. (38)
**Dabigatran**			
ECA-test	*NA*	1.00	Groene et.al. (29)
	0 to 375	1.00	Oberladstätter et al. (38)

DOAC plasma concentration presents as median and interquartile range, mean and standard deviation, or total range. Coefficients refer to correlation test of the original study either to Spearman’s, Pearson’s, or regression analysis. RVV-test, Russell’s viper venom activated test; ECA-test, ecarin activated test.

##### Viscoelastic thresholds for apixaban concentrations

There is no result with ROTEM EXTEM and INTEM for apixaban thresholds detection. Apixaban plasma concentrations of ≥30 and of ≥50 ng/ml can be detected by threshold values of the AFXa channel by TEG 6s and RVV-test by ClotPro (20–22, 38) (Figure 5 and Tables 1.1–1.3). No differences in R times between apixaban peak and through samples were found in another study (25).

#### Edoxaban

Six studies (25, 29, 38, 45, 46, 55) were identified analyzing the effects if edoxaban on viscoelastic testing (Supplementary Tables 2.1–2.3). The CT and R in relation to the edoxaban plasma concentration are shown in Figure 4.

##### Edoxaban and rotational thrombelastometry

By the time literature review was concluded there were only two studies assessing the effects of edoxaban on ROTEM showing significant prolongations of EXTEM clotting time (CT) even in therapeutic doses (45, 55) (Table 2.1).

##### Edoxaban and thrombelastography

Two studies described effects of edoxaban on the anti-factor-Xa channel [AFXa] of the TEG 6s and TEG 5000 system (Supplementary Table 2.2). The one study reported a pooled analysis of various DOACs together, not allowing for an individual result analysis (56). The other reported a change in parameters at doses several times higher than peak plasma levels (46) (Table 2.1).

##### Edoxaban and ClotPro analyzer

Alterations of ClotPro parameters by edoxaban were found in two studies (Supplementary Table 2.3). Main changes occurred to CT of the russel viper venom test, showing high sensitivity and specificity for a low threshold (38). Further, a statistically significant prolongation of the CT for this test was only seen in patients taking edoxaban compared to the other anti-factor Xa inhibitors rivaroxaban and apixaban (29) (Tables 1.3, 2.3).

##### Viscoelastic thresholds for edoxaban concentrations

Edoxaban plasma concentrations of 50 and 100 ng/ml can be detected by threshold values of clotting time (CT) (38) (Figure 5 and Table 1.2).

### Viscoelastic analysis of direct factor II inhibitor

#### Dabigatran

29 studies described dabigatran action in whole blood with viscoelastic methods (20–22, 25–27, 29–31, 34, 35, 38, 45, 48, 57–71) (Supplementary Tables 2.1–2.3). As already mentioned for the factor Xa inhibitors, dabigatran showed either no significant influence or was not analyzed for other parameters except for the viscoelastic parameters clotting time (CT) and reaction time (R). The CT and R in relation to the dabigatran plasma concentration are shown in Figure 4.

##### Dabigatran and rotational thrombelastometry

Fourteen studies assessed dabigatran in whole blood with ROTEM (27, 30, 31, 45, 48, 58, 60, 61, 68–71) (Supplementary Table 2.1). Out of these, one study did not report the exact dabigatran concentration under which conditions the viscoelastic tests were performed (48). Most studies analyze the EXTEM (27, 30, 31, 34, 45, 58, 60, 64, 68–70) and INTEM (27, 30, 31, 34, 45, 58, 60, 64, 68, 70) original assays, with less data published on NATEM (61, 68) and FIBTEM (34, 45, 58, 70). Four studies reported results obtained from *ad hoc* designed or modified original assays (48, 60, 69, 70).

The majority of studies describe a correlation between plasma dabigatran concentrations with a linear increase of clotting time (CT), some highlighting a higher sensitivity of EXTEM over INTEM (27, 31, 34, 45, 58, 64, 68, 70, 72). In detail, a significant correlation between dabigatran plasma concentration and duration of clotting time (CT) varied from strong [EXTEM (34), INTEM (34, 70)] to very strong [EXTEM (45, 58, 64, 70), INTEM (45, 58, 60, 64) and FIBTEM (58, 70)] (Table 2.1).

##### Dabigatran and thrombelastography

Four studies investigated dabigatran action with the new cartridge-based direct thrombin inhibitor [DTI] channel of TEG 6s (20–22, 25), to our knowledge not yet commercially available at the time of manuscript preparation. Of these, two (22, 25) did not report the exact dabigatran concentration under which conditions the viscoelastic tests were performed.

The further studies investigated parameters with the TEG 5000 system (26, 31, 35, 57, 62, 63, 65–67) (Supplementary Table 2.2).

Dabigatran treated whole blood led to a significant prolongation of reaction time (R) when compared to baseline or control values in regard to the direct thrombin inhibitor channel (20), the citrated kaolin channel (26, 35, 67, 73), RapidTEG*™* channel (26, 62), and calcium chloride (CaCl_2_) channel (63). One study did not find any effect of dabigatran on TEG 5000 parameters (57).

A significant correlation between dabigatran plasma concentration and reaction time (R) was shown for the direct thrombin inhibitor channel [very strong correlation (20, 21)], for the citrated kaolin channel [strong to very strong correlation (35, 65, 67)], and calcium chloride channel [moderate correlation (63)] (Table 2.2).

##### Dabigatran and ClotPro analyzer

The three studies performed with ClotPro showed moderate (59) to very high (29, 38) correlation between plasma dabigatran concentration and clotting time (CT) of pathway, specific for the detection of factor IIa antagonist (Supplementary Table 2.3).

##### Viscoelastic thresholds for dabigatran concentrations

Dabigatran plasma concentrations between 20 and 100 ng/ml can be detected by threshold values of the viscoelastic parameters CT and R (20–22, 30, 38, 69) (Figure 5; Tables 1.1–1.3). In particular, the ECA-test of ClotPro and the DTI channel of TEG 6s show consistent statistical sensitivity and specificity.

### Impact of andexanet alfa and idarucizumab on viscoelastic testing

The specific antagonists andexanet alfa and idarucizumab have been developed for the reversal of direct oral anticoagulants. There is limited to no published information on viscoelastic coagulation testing for specific DOAC reversal (3) despite this method being of particular importance, as the treatment monitoring after administration of andexanet alfa should not be based on commercial anti-FXa activity assays (74, 75). In these assays, the FXa inhibitor dissociates from andexanet alfa resulting in the detection of falsely elevated anti-FXa activity levels.

We found two studies reporting viscoelastic testing after the specific reversal of DOAC (68, 76). No data is available for ROTEM on behalf of andexanet alfa. Takeshita et al. investigated the reversal of dabigatran by adding idarucizumab, which resulted in both INTEM and EXTEM clotting time reversal toward reference ranges (68). Oberladstätter et al. investigated the specific reversal of dabigatran with ClotPro ecarin clotting time (ECA-test CT) and apixaban, edoxaban, and rivaroxaban with ClotPro Russell’s viper venom test clotting time (RVV-test CT) (76). Idarucizumab substantially reduced ECA-CT, whereas andexanet alfa did not normalize the RVV-CT. Andexanet alfa spiking of non-anticoagulated blood prolonged RVV-CT, potentially as a consequence of a competitive antagonism with human factor Xa.

## Discussion

This review shows the effect of the DOACs rivaroxaban, apixaban, edoxaban, and dabigatran on viscoelastic point-of-care tests. A total of 53 studies were included and qualitatively analyzed. Mainly, studies report ROTEM and TEG measurement methods, with rivaroxaban and dabigatran being the most studied.

### Correlation of direct oral anticoagulant plasma concentration with viscoelastic tests

Direct oral anticoagulants (DOACs) show a clear influence on CT and R, resulting in being the main focus of studies. Other ROTEM and TEG parameters (e.g., MCF, A10, LI 60, or alpha angle) were either not further analyzed or showed no to minor changes in the reported studies. By using different activators, viscoelastic tests distinguish extrinsic, intrinsic or total pathways of coagulation in relation to the DOAC effect. The most specific results are produced by viscoelastic assays that reflect thrombin generation by measuring the physiological constitutional change of blood from the viscous to the clotted state. In principle, this reflects the length of CT as well as R. Accordingly, most significant concentration-dependent changes are described for ROTEM INTEM/EXTEM-CT and for TEG AFXa/DTI as well as CK channel R time. Overall, the results of the analyzed studies were trending toward DOACs showing a higher correlation of CT with drug concentration in the EXTEM channel over INTEM and FIBTEM. With TEG, the greatest affinity resulted with the AFXa or DTI channel, which are currently not yet available. Stronger correlations were demonstrated in assays with alternative not commercially available activators, but these are isolated examples and beyond the scope of this review. Not only is it important to consider the right cartridge and channel, but also the mechanism of action of the DOAC, distinguishing between dabigatran and factor-Xa inhibitors. In regards to the latter, the two most analyzed drugs, rivaroxaban and apixaban, show distinct differences in their affinity, even in identical conditions, potentially explaining the discrepancies in CT with apixaban (77). ROTEM tests were only poorly impacted by low levels of rivaroxaban, edoxaban or dabigatran, and apixaban had only a low effect even at high concentrations.

### Detection of clinically relevant direct oral anticoagulant plasma concentrations with viscoelastic tests

Of particular interest are threshold values of clotting time CT or reaction time R at which a certain DOAC concentration must be assumed. It was shown that a cut-off value of 50 ng/ml does not exacerbate ongoing hemorrhage in bleeding patients (78, 79). For surgery with high bleeding risk, a preoperative DOAC concentration less than 30 ng/ml is proposed (78, 80, 81). In surgery with high expected blood loss, a calculated rivaroxaban concentration of greater than 100 ng/ml was associated with a significant increase of perioperative red blood cell loss (82). For thrombolysis in patients with acute ischemic stroke, plasma concentrations up to 100 ng/ml have been suggested to be acceptable (83). According to current guidelines, administration of reversal agents in bleeding patients on DOACs should be considered if plasma concentrations exceed 50 ng/ml (80). Most of the research regarding perioperative bleeding thresholds focuses on rivaroxaban, as this is the most prescribed DOAC (79, 81). We are not aware of any study investigating the interchangeability of above mentioned perioperative bleeding thresholds between different DOACS.

Most data are available for rivaroxaban and dabigatran. We revealed a good sensitivity of viscoelastic parameters in patients using DOAC and might therefore be a good candidate for emergency testing. The added advantage is that the results are readily available and there is uniform performance. But it must be assumed that viscoelastic methods are not sensitive enough to determine specific DOAC concentrations. Results within the normal reference range do not reliably exclude relevant residual DOAC plasma levels and limit their clinical implications. Further, traditional viscoelastic coagulation monitoring assays were not designed to measure the effects of DOACs. Of interest, the costs of DOAC specific laboratory measurements, such as anti-Xa-activity or liquid-chromatography mass-spectrometry (a more accurate method compared to HP-LC) are $40USD and $130USD, with a turnaround time of approximately 30–60 min and 2-4 h, respectively. Moreover, liquid-chromatography mass-spectrometry measurements may not be available 24/7. Viscoelastic tests cost on average about $70USD per analysis and provide first results within minutes. However, there may be price differences depending on the manufacturer and country-specific health system.

### Monitoring the specific direct oral anticoagulant reversal

The use of commercially available anti-FXa assays to measure rivaroxaban or apixaban concentrations in patients after reversal with andexanet alfa has limitations. One of the limitations is the large sample dilution in the assay set-up, which causes dissociation of the inhibitor from the andexanet alfa-inhibitor complex, resulting in an erroneous elevation of the anti-FXa activity (5). For rivaroxaban, the residual drug concentration 4 h after treatment with andexanet alfa was approximately 42% lower than the pretreatment concentration (84). A concentration that can still affect hemostasis. Thus, viscoelastic testing may play an important role in monitoring after andexanet alfa reversal (85).

For dabigatran reversal, there appears to be a rebound or dissociation effect after 12 to 24 h (5). Measurements of dabigatran may predict the need for secondary dosing of this reversal agent. In a retrospective study, it has been shown that no plasma dabigatran rebound was observed after reversal in patients with dabigatran plasma level <264 ng/mL at baseline (86). Further, in a case of ongoing bleeding by chronic accumulation of dabigatran showed impressively ongoing redistribution of dabigatran necessitates repetitive application of idarucizumab to neutralize dabigatran (87). Accordingly, repeated and timely coagulation monitoring is required.

### Limitation

The studies are heterogeneous, and their replication of results was not constant. For apixaban in particular, there are heterogeneous data for the effect on CT EXTEM in ROTEM. This may be attributed to methodological differences in the work of Escolar et al. and Pujadas-Mestres et al. (49, 51). Most studies are based on a small study population or sample collection. The analyses reflect different populations including healthy volunteers with spiked samples and *in vitro* analyses. Furthermore, there is a lack of international reference values among the different tests. On the other hand, this work includes a large number of studies. Direct conclusions for the treatment of patients under DOAC can be made for clinical use from this review. We omitted betrixaban from our review as the drug was discontinued by the manufacturer in April 2020 for independent business reasons and never received approval from the European Medicines Agency.

## Conclusion

Viscoelastic test assays can provide fast and essential point-of-care information regarding residual DOAC activity, especially DOAC specific assays. Even with strong correlation between the DOAC plasma concentration and viscoelastic parameter clotting time (CT) or reaction time (R), the results could be within the normal reference range. The quantification of residual DOAC plasma concentration with DOAC unspecific viscoelastic assays is not sensitive enough, compared with recommended anti-Xa activity laboratory measurements.

## Data availability statement

The original contributions presented in the study are included in the article/Supplementary material, further inquiries can be directed to the corresponding author.

## Author contributions

SDS, DRS, and AK contributed to conception and design of the study. SDS and CC organized the database. SDS wrote the first draft and processed the manuscript. CC wrote sections of the manuscript. All authors contributed to manuscript revision, read, and approved the submitted version.
