# Supplementary material for: The impact of direct oral anticoagulants on viscoelastic testing – A systematic review
Source: Front Cardiovasc Med. 2022 Nov 7;9:991675. doi: 10.3389/fcvm.2022.991675 (PMC9676657; doi:10.3389/fcvm.2022.991675)
Supplement: Supplementary file 1 [file Data_Sheet_1.docx]

Supplementary Material for:

**The Impact of Direct Oral Anticoagulants on Viscoelastic testing**

**– A Systematic Review**

Content of additional tables:

**- Supplementary** **Table 1:** Risk of Bias Assessment using the Newcastle-Ottawa Quality Assessment Scale for cohort studies (http://www.ohri.ca/programs/clinical_epidemiology/oxford.asp) where a higher number of stars equals a better quality.

**- Supplementary** **Table 2.1:** Details of included studies for ROTEM®.

**- Supplementary** **Table 2.2:** Details of included studies for TEG®.

**- Supplementary** **Table 2.3:** Details of included studies for ClotPro®.

**Supplementary** **Table 1:** Risk of Bias Assessment using the Newcastle-Ottawa Quality Assessment Scale for cohort studies (http://www.ohri.ca/programs/clinical_epidemiology/oxford.asp) where a higher number of stars equals a better quality.

|  | Selection | | | | Comparability | Outcome | | |  |
| --- | --- | --- | --- | --- | --- | --- | --- | --- | --- |
| **Reference** | **Representativeness of the exposed cohort** | **Selection of non-exposed cohort** | **Ascertainment of exposure** | **Outcome not present at start** | **Comparability of controls** | **Assessment of outcome** | **Adequate follow up** | **Loss to follow up** | **Total score** |
| Adelmann et. al. 2014 ^1^ | * | - | * | * | - | * | * | * | 6/9 |
| Aho et. al. 2016 ^2^ | * | - | * | * | - | * | * | * | 6/9 |
| Aranda et. al. 2019 ^3^ | * | - | * | * | - | * | * | * | 6/9 |
| Artang et. al. 2021 ^4^ | * | - | * | * | - | * | * | * | 6/9 |
| Artang et. al. 2019 ^5^ | - | - | * | * | - | * | * | * | 5/9 |
| Bliden et. al. 2017 ^6^ | * | - | * | * | - | - | * | * | 5/9 |
| Casutt et. al. 2012 ^7^ | - | - | * | * | - | * | * | * | 5/9 |
| Chojnowski et. al. 2015 ^8^ | * | - | * | * | - | * | * | * | 6/9 |
| Comuth et. al. 2018 ^9^ | * | - | * | * | - | * | * | * | 6/9 |
| Dias et. al. 2019 ^10^ | * | - | * | * | - | - | * | * | 5/9 |
| Dias et. al 2015 ^11^ | - | - | * | * | - | * | * | * | 5/9 |
| Eller et. al. 2014 ^12^ | - | - | * | * | - | * | * | * | 5/9 |
| Escolar et. al. 2013 ^13^ | - | - | * | * | - | * | * | * | 5/9 |
| Fong et. Al. 2020 ^14^ | * | - | * | * | - | * | * | * | 6/9 |
| Fontana et. al. 2017 ^15^ | - | - | * | * | - | - | * | * | 4/9 |
| Groene et.al. 2021 ^16^ | * | - | * | * | - | - | * | * | 5/9 |
| Havrdová et. al. 2020 ^17^ | - | - | * | * | - | * | * | * | 5/9 |
| Henskens et. al. 2018 ^18^ | * | - | * | * | - | * | * | * | 6/9 |
| Herrmann et. al. 2014 ^19^ | * | - | * | * | - | * | * | * | 6/9 |
| Jenrette et. a. 2022 ^20^ | * | - | * | * | - | * | * | * | 6/9 |
| Kaaber et. al. 2021 ^21^ | * | - | * | * | - | * | * | * | 6/9 |
| Klages et. al. 2021 ^22^ | * | - | * | * | - | * | * | * | 6/9 |
| Kopytek et. al. 2020 ^23^ | * | - | * | * | - | * | * | * | 6/9 |
| Körber et. al. 2017 ^24^ | * | - | * | * | - | * | * | * | 6/9 |
| Körber et. al. 2014 ^25^ | - | - | * | * | - | * | * | * | 5/9 |
| Kyriakou et. al. 2018 ^26^ | * | - | * | * | - | * | * | * | 6/9 |
| Kyriakou et. al. 2015 ^27^ | * | - | * | * | - | * | * | * | 6/9 |
| Myers et. al. 2020 ^28^ | * | - | * | * | - | * | * | * | 6/9 |
| Nadtochiy et. al. 2020 ^29^ | - | - | * | * | - | * | * | * | 5/9 |
| Oberladstätter et. al. 2020 ^30^ | * | - | * | * | - | * | * | * | 6/9 |
| Oswald et. al. 2015 ^31^ | * | - | * | * | - | * | * | * | 6/9 |
| Pailleret et. al. 2019 ^32^ | - | - | * | * | - | * | * | * | 5/9 |
| Perzborn et. al. 2014 ^33^ | - | - | * | * | - | * | * | * | 5/9 |
| Pipilis et. al. 2017 ^34^ | * | - | * | * | - | * | * | * | 6/9 |
| Pujadas-Mestres et. al. 2017 ^35^ | - | - | * | * | - | * | * | * | 5/9 |
| Rathburn et. al. 2015 ^36^ | * | - | * | * | - | * | * | * | 6/9 |
| Samama et. al. 2010 ^37^ | - | - | * | * | - | * | * | * | 5/9 |
| Schenk et. al. 2016 ^38^ | - | - | * | * | - | * | * | * | 5/9 |
| Schmidt et. al. 2019 ^39^ | * | - | * | * | - | * | * | * | 6/9 |
| Seyve et. al. 2018 ^40^ | - | - | * | * | - | * | * | * | 5/9 |
| Siddiqui et. al. 2019 ^41^ | - | - | * | * | - | * | * | * | 5/9 |
| Sokol et. al. 2021 ^42^ | * | - | * | * | - | * | * | * | 6/9 |
| Solbeck et. al. 2018 ^43^ | * | - | * | * | - | * | * | * | 6/9 |
| Solbeck et. al. 2016 ^44^ | - | - | * | * | - | * | * | * | 5/9 |
| Solbeck et. al. 2014 ^45^ | - | - | * | * | - | - | * | * | 4/9 |
| Spinthakis et. al. 2019 ^46^ | * | - | * | * | - | - | * | * | 5/9 |
| Takeshita et. al. 2020 ^47^ | - | - | * | * | - | * | * | * | 5/9 |
| Taune et. al. 2018 ^48^ | * | - | * | * | - | * | * | * | 6/9 |
| Taune et. al. 2017 ^49^ | * | - | * | * | - | * | * | * | 6/9 |
| Tsantes et. al. 2015 ^50^ | * | - | * | * | - | * | * | * | 6/9 |
| Tsantes et. al. 2016 ^51^ | * | - | * | * | - | * | * | * | 6/9 |
| Vedovati et. al. 2020 ^52^ | * | - | * | * | - | - | * | * | 5/9 |
| Voukalis et. al. 2021 ^53^ | * | - | * | * | - | - | * | * | 5/9 |

For ‘Representativeness’ we awarded one star if patients taking DOACs were included and none if blood from healthy volunteers was spiked with a DOAC. Regarding ‘Selection of Non-Exposed Cohort’, a star was given if samples were taken from the same community as the exposed cohort. Healthy volunteers did not fulfill this criterion. The requirement ‘Ascertainment of Exposure’ is fulfilled if data are collected from safe records. If the outcome was not available initially, we added one star. For the category ‘Comparability of controls’ up to two stars could be awarded for the study’s most important and one additional factor. When authors documented a DOAC concentration and the viscoelastic assay, we added one start to ‘Assessment of Outcome’. An adequate follow-up is provided when the duration of the viscoelastic test is long enough to retrieve all required parameters. We added one star when studies reported a complete follow-up or only a small number was lost and unlikely to cause bias.

**Supplementary** **Table 2.1:** Details of included studies for ROTEM®.

| **Author, Year, and Country** | **Journal** | **Study design** | **Population** | **DOAC** | **Assay** | **Plasma concentration of DOAC (ng/mL)** | **Main result** |
| --- | --- | --- | --- | --- | --- | --- | --- |
| Adelmann et. al. 2014, Austria ^1^ | Thromb Res | ex vivo and observational | 20 volunteers, 20 patients on rivaroxaban, 20 on apixaban and 20 controls | Apixaban Rivaroxaban | LowTF PiCT | R: 60 (± 20), 160 (± 10), 200 (± 20), 290 (±30), 420 (± 30); patient samples 535 (± 147); A: 50 (± 10), 120 (± 10), 250 (± 30), 300 (±30), 420 (± 20); patient samples 64 (± 56); mean (SE) | LowTF modified ROTEM® could be a valuable diagnostic tool for rapid DOAC determination. |
| Aranda et. al. 2019, Brazil ^3^ | Braz J Med Biol Res | interventional | 64 clinically stable patients | Rivaroxaban | NATEM | trough 18.3 (± 30.7) and peak 185.1 (± 65.4); mean (SE) | Positive correlation (at peak) between the plasma concentration and CT, CFT. CT is a good predictors of rivaroxaban plasma concentration > 30 ng/mL. |
| Casutt et. al. 2012, Switzerland ^7^ | Anaesthesist | interventional | 11 healthy male volunteers | Rivaroxaban | INTEM EXTEM | *NA* | Significant differences compared to the baseline values were found in the EXTEM-CT and INTEM-CT. Rivaroxaban treated patients could still show normal ROTEM® values. |
| Chojnowski et. al. 2015, Poland ^8^ | Adv Clin Exp Med | observational | 13 patients with venous thromboembolism (20mg) and 13 healthy volunteers for reference ranges | Rivaroxaban | INTEM EXTEM | 152.6 (107.2 to 197.9) mean (95% CI); SD 75.0 | EXTEM-CT may be used to determine the anticoagulation effect of rivaroxaban, but is not sensitive enough to measure the residual activity of this drug. |
| Comuth et. al. 2018, Denmark ^9^ | Thromb Res | ex vivo & observational | 10 healthy donors and samples from 15 patients treated for AF | Dabigatran | INTEM EXTEM FIBTEM | 0, 25, 50, 100, 250, 500, 1000 spiked; patients value 0 to 833 | EXTEM-CT, INTEM-CT, and FIBTEM-CT correlate well with dabigatran plasma concentrations. Storage of dabigatran plasma samples at −80 °C for up to 12 months does not influence measured levels. |
| Eller et. al. 2014, Germany ^12^ | Clin Chem Lab Med | ex vivo | 10 healthy volunteers | Apixaban Rivaroxaban Dabigatran | INTEM EXTEM | 100, 200, 300, 500, 2000 spiked | Apixaban and rivaroxaban exhibit effects on CT. Dabigatran (100 to 300 ng/ml) significantly changed EXTEM-CT and INTEM-CT. |
| Escolar et. al. 2013, Spain ^13^ | PLoS One | ex vivo | 10 healthy volunteers | Apixaban | EXTEM | 200 spiked | Apixaban significantly prolonged clotting times (CTs) in TEM studies. |
| Fontana et. al. 2017, Switzerland ^15^ | Thromb Res | interventional multicenter validation study | 20 healthy volunteers | Rivaroxaban | INTEM EXTEM FIBTEM HEPTEM | *NA* | No precise quantification of plasma concentration. EXTEM-CT detect the presence of rivaroxaban at peak level with a high sensitivity. Rivaroxaban 20 mg OD treatment significantly alters ROTEM® parameters. |
| Havrdová et. al. 2020, Finland ^17^ | J Clin Pharmacol | observational | 15 healthy male volunteers | Edoxaban | EXTEM FIBTEM | 0 to 500 spiked approximately | CT correlates significantly with edoxaban plasma concentrations and can be used to estimate the effect of edoxaban. |
| Henskens et. al. 2018, The Netherlands ^18^ | Thromb J | observational | R: 109 and D: 75 patients with non-valvular atrial fibrillation | Rivaroxaban Dabigatran | INTEM EXTEM | R: 187 (± 139) and D: 104 (± 53); mean (SE) | EXTEM-CT and INTEM-CT may be fast whole blood alternatives to detect the presence of significant plasma levels. |
| Herrmann et. al. 2014, Australia ^19^ | Thromb Haemost | observational | R: 15 patients for the prevention of deep venous thrombosis after hip or knee replacement surgery; D: 17 patients with NVAF | Rivaroxaban Dabigatran | INTEM EXTEM | R: 133.0 (104.6 to 169.0) and D: 128.6 (81.1 to 204.0); mean (95% CI) | Rivaroxaban did not affect the ROTEM parameters compared to the normal range. For dabigatran, EXTEM-CFT and INTEM-CFT prolonged to reference range with significant correlation. |
| Klages et. al. 2021, Germany ^22^ | Minerva Anestesiol | prospective | R: 16 and D: 16 patients with rivaroxaban treatment | Rivaroxaban Dabigatran | INTEM EXTEM FIBTEM | R: trough 84.2 (20.24 to 340.7); peak 206.4 (43.4 to 350.4) and D. trough 33.5 (0 to 227.7); peak 82.3 (17.8 to 251.6) | EXTEM-CT showed a significant positive correlation with plasma levels and EXTEM-CT seems to facilitate qualitative monitoring of dabigatran. |
| Körber et. al. 2014, Germany ^25^ | Clin Appl Thromb Hemost | ex vivo | 10 healthy adult volunteers | Rivaroxaban | EXTEM | 100 (94 to 104), 231 (217 to 262); median (25 to 75% quantiles) | Rivaroxaban increased EXTEM-CT dose dependently. |
| Körber et. al. 2017, Germany ^24^ | Transfus Med Hemother | observational | Ten patients undergoing total hip or knee arthroplasty | Dabigatran | INTEM EXTEM ECATEM | 107 (91 to 305), 47 (28 to 147), and 9 (0 to 59); median (25 to 75% quantiles) | Only INTEM-CT and ECATEM-CT correlated significantly with the measured dabigatran concentration. ECATEM-CT appears a valid POC method parameter to detect thrombin inhibition. |
| Kyriakou et. al. 2015, Greece ^27^ | Clin Appl Thromb Hemost | observational | 20 patients with NVAF vs matched Marcoumar | Dabigatran | NATEM | 956 (761 to 1150); median (IQR) | No correlation of ROTEM parameters with dabigatran plasma level. |
| Kyriakou et. al. 2018, Greece ^26^ | Clin Appl Thromb Hemost | observational | 20 patients with NVAF and 20 controls | Apixaban | NATEM | 223.5 (147 to 329); median (IQR) | Significant prolonged CT compared to control but no correlation to plasma concentrations. |
| Oswald et. al. 2015, Austria ^31^ | Blood Coagulation and Fibrinolysis | observational | 61 patients scheduled for major orthopedic surgery | Rivaroxaban | INTEM EXTEM | *NA* | Significant prolongation of the parameters CT, CFT, and MCF compared to baseline. |
| Pailleret et. al. 2019, France ^32^ | Eur J Anaesthesiol | ex vivo and observational | 30 patients on rivaroxaban, 17 on apixaban and 19 without treatment | Apixaban Rivaroxaban | Modified-R | 0, 25, 100, 200 spiked; patient samples 20 to 483 | Modified ROTEM may be applicable in emergency situations for the detection of FXa inhibitors in whole blood. Various triggering conditions were examined in variations of plasma constitutions. |
| Perzborn et. al. 2014, Germany ^33^ | Thromb Res | ex vivo | *NA* healthy volunteers | Rivaroxaban | EXTEM | 0, 200, 500, 1000 spiked | Rivaroxaban plasma concentrations between 331 and 1807 ng/mL concentration dependently prolonged EXTEM-CT from 1.8-fold to 3.7-fold over baseline. |
| Pujadas-Mestres et. al. 2017, Spain ^35^ | PLoS One | ex vivo | healthy donors | Apixaban | EXTEM | 0, 10, 40 and 160 spiked | Apixaban significantly prolonged the parameters CT and CFT. These prolongations reached levels of statistical significance for concentrations of apixaban > 40 ng/mL. |
| Schenk et. al. 2016, Austria ^38^ | Br J Anaesth | ex vivo | 13 healthy volunteers and 20 patients treated with rivaroxaban | Rivaroxaban | EXTEM FIBTEM | 0, 100, 200, 300, 400, 500, 600, 700 spiked | Significant, rivaroxaban dependent prolongation and a clinically relevant increase in EXTEM-CT and FIBTEM-CT compared with normal values. |
| Schmidt et. al. 2019, Germany ^39^ | Blood Transfus | observational | 10 patients with elective knee or hip replacement surgeries | Apixaban | EXTEM | 77 (41 to 95), 92 (62 to 104), 59 (38 to 95); mean (IQR) | Apixaban caused a statistically significant prolongation of EXTEM-CT. |
| Seyve et. al. 2018, France ^40^ | Int J Lab Hematol | ex vivo | 5 healthy donors each | Apixaban Edoxaban Rivaroxaban Dabigatran | INTEM EXTEM FIBTEM | 0, 50, 100, 200, 500, 1000 spiked | Linear relationship between the increase in CT and plasma DOAC concentrations in both the EXTEM and INTEM tests. Only poorly impacted by low levels. Apixaban had only a low effect even at high concentrations. |
| Sokol et. al. 2021, Slovakia ^42^ | Clin Appl Thromb Hemost | observational | 27 patients treated with dabigatran 150 mg BID due to NVAF | Dabigatran | INTEM EXTEM | trough 74 (11.2 to 250); post-dose 120 (31 to 282) | EXTEM-CT and INTEM-CT has a strong and highly significant correlation with the plasma dabigatran concentration. |
| Takeshita et. al. 2020, Japan ^47^ | Anesth Analg | ex vivo | 12 healthy volunteers | Dabigatran | INTEM EXTEM | 0, 200, 500, 1000, 1500, 2000, 3000, 5000 spiked | Dabigatran prolonged EXTEM-CT and INTEM-CT in a concentration-dependent manner. |
| Taune et. al. 2017, Sweden ^49^ | Thromb Res | observational | 30 patients on dabigatran 150 mg BID | Dabigatran | INTEM EXTEM FIBTEM LowTF | 86 (29 to 150) and 175 (67 to 490); median (IQR) | EXTEM-CT and FIBTEM-CT showed a strong correlation with dabigatran concentrations in real-life atrial fibrillation patients. |
| Taune et. al. 2018, Sweden ^48^ | J Thromb Haemost | ex vivo and observational | 10 healthy donors and 35 patients taking dabigatran because of AF | Dabigatran | EXTEM thrombin-based trigger (*E12) | 0, 20, 50, 100, 300, and 500 spiked | ROTEM-CT with a thrombin-based trigger (*E12) is more sensitive to dabigatran effects than EXTEM-CT, and detects anticoagulant effects of drug concentrations in the low to very low therapeutic range. |
| Tsantes et. al. 2015, Greece ^50^ | J Neurol Sci | prospective | 19 patients with cerebrovascular diseases and NVAF | Dabigatran | NATEM | 70 (35 to 140); median (IQR) | Dabigatran treatment led to a reduction of the LI60 in univariate analysis. |
| Tsantes et. al. 2016, Greece ^51^ | Medicine | observational | 20 patients with NVAF and 20 control patients | Rivaroxaban | NATEM | 200 (85 to 345); median (IQR) | All indices (except MCF and LI 60) were influenced compared to control. But no correlation with rivaroxaban level. |
| Vedovati et. al. 2020, Italy ^52^ | J Thromb Thrombolysis | observational | 10 patients with NVAF and 15 healthy volunteers | Apixaban Rivaroxaban Dabigatran | A, R: diluted-EXTEM a-FXa catcher D: ECATEM-B a-FIIa catcher | *NA* peak and trough | Ad-hoc designed reagents were able to accurately identify apixaban, rivaroxaban, and dabigatran activity. |

**Abbreviations** - Assays: **NATEM** non-activated rotational thromboelastometry; **EXTEM** extrinsic activated rotational thromboelastometry; **INTEM** intrinsic activated rotational thromboelastometry; **HEPTEM** intrinsic activated rotational thromboelastometry with added heparinase; **FIBTEM** extrinsic activated rotational thromboelastometry with added cytochalasin D; Modified assays customized by study team; **LowTF** assay activated with low tissue factor; **PiCT** prothrombinase induced clotting time reagent; **ECATEM** uses ecarin to initiate rotational thromboelastometry; **m-ROTEM** assay activated with tissue factor and phospholipid vesicles; **Thrombin-b** assay activated with thrombin-based trigger. Parameters - **CT** clotting time; **CFT** clot formation time; **MCF** maximum clot firmness; **LI60** lysis index after 60 minutes.

**DOAC** direct oral anticoagulant; **R** rivaroxaban; **A** apixaban; **D** dabigatran; **AF** atrial fibrillation; **NVAF** non valvular atrial fibrillation; **NA** not applicable; **OD** once a day; **BID** twice a day; **POC** point-of-care; **CI** confidence interval; **IQR** interquartile range; **SD** standard deviation, **SE** standard error.

**Supplementary** **Table 2.2:** Details of included studies for TEG®.

| **Author, Year, and Country** | **Journal** | **Study design** | **Population** | **DOAC** | **Assay** | **Plasma concentration of DOAC (ng/mL)** | **Main result** |
| --- | --- | --- | --- | --- | --- | --- | --- |
| Aho et. al. 2016, New Zealand ^2^ | Anaesth Intensive Care | observational | 20 patients with NVAF | Dabigatran | TEG®5000 Kaolin-TEG | 71 (39 to 98); median (IQR) | No consistent effect of dabigatran on the TEG. |
| Artang et. al. 2021, U.S.A. ^4^ | TH Open | observational | 53 patients on rivaroxaban, 50 on apixaban, and 62 on dabigatran; 24 controls | Apixaban Rivaroxaban Dabigatran | TEG®6s AFXa | 29 to 99 | R-time measured by TEG®6s DOAC-specific cartridge has a strong correlation with concentrations of the most commonly used DOACs. |
| Artang et. al. 2019, U.S.A. ^5^ | Res Pract Thromb Haemost | interventional | 9 healthy males | Apixaban Rivaroxaban Dabigatran | TEG®6s AFXa DTI | A: 104.2 (73.8 to 144.5); R: 205.5 (94.2 to 317.9); D: 92.1 (40.7 to 196.9); median (total range) | Associated with 100% sensitivity and ≥ 90% specificity to detect DOAC levels of ≥ 50 ng/mL. No correlations between other TEG parameters and DOAC concentrations. |
| Bliden et. al. 2017, U.S.A. ^6^ | J Thromb Thrombolysis | observational | 16 patients on apixaban, 24 on rivaroxaban and dabigatran; patients with concomitant ASS and P2Y12-inhibitor therapy | Apixaban Rivaroxaban Dabigatran | TEG®6s AFXa DTI | *NA* | TEG®6s R-time highly correlated to rivaroxaban concentration. The automated TEG®6s DTI assay may be an effective tool to identify an anticoagulant effect. Cut-off time is calculated in a pooled setting. |
| Dias et. al. 2019, Switzerland ^10^ | J Trauma Acute Care Surg | observational, prospective, multicenter | 54 patients on apixaban, 4 on edoxaban, 57 on rivaroxaban, and 75 on dabigatran; Reference range population n=160); non-DOAC population n=24 | Apixaban Edoxaban Rivaroxaban Dabigatran | TEG®6s AFXa DTI | *NA*  trough and non-trough | TEG®6s can detect and classify DOACs with high sensitivity and specificity with algorithm. States reference ranges for R time. |
| Dias et. al 2015, Switzerland ^11^ | Arch Pathol Lab Med | ex vivo | 3 healthy volunteers, 1 control | Apixaban Rivaroxaban Dabigatran | TEG®5000 Kaolin-TEG RapidTEG^TM^ +/- ecarin | A: 250, 500, 1000; R: 22, 89, 500; D: 50, 200, 500 spiked | The RapidTEG activated clotting time test and the kaolin test appear to be capable of detecting and monitoring DOAC. The ecarin test may be used to differentiate between Xa inhibitors and direct thrombin inhibitors. |
| Herrmann et. al. 2014, Australia ^19^ | Thromb Haemost | observational | 17 patients with NVAF | Rivaroxaban Dabigatran | TEG®5000 *NA* | R: 133.0 (104.6 to 169.0) D: 128.6 (81.1 to 204.0); mean (95% CI) | Rivaroxaban did not affect the TEG parameters. Dabigatran significantly increased the TEG parameters (R, alpha angle) without significant correlation. |
| Jenrette et. a. 2022, U.S.A. ^20^ | Am J Emerg Med | observational | 21 patients on rivaroxaban, 19 on apixaban in an emergency department trauma population | Apixaban Rivaroxaban | TEG®5000 Kaolin-TEG | *NA* | TEG parameters are not consistently affected by use of DOAC therapy in an emergency department trauma patient population. |
| Kaaber et. al. 2021, Denmark ^21^ | Scand J Clin Lab Invest | observational | 159 patients with rivaroxaban, and 184 with apixaban where the hemostasis management on call team was contacted | Apixaban Rivaroxaban | TEG®5000 RapidTEG^TM^ | *NA* | Those with a TEG-ACT above normal reference had a significantly increased risk of severe bleeding with high transfusion demands. |
| Kopytek et. al. 2020, Poland ^23^ | J Physiol Pharmacol | observational | A, R: 20 and D: 13 venous thrombo-embolism patients | Apixaban Rivaroxaban | TEG®5000 Kaolin-TEG | A: 85 (40 to 105); R: 99 (48 to 311); D: 71 (39 to 98); median (IQR) | Plasma concentrations correlated with Ks and CLT. For dabigatran, fibrin clot properties correlate with corresponding TEG indices. |
| Myers et. al. 2020, U.S.A. ^28^ | Anesthesiology | observational | 80 trauma patients with preinjury rivaroxaban use within 48 h of presentation and 20 controls | Rivaroxaban | TEG®5000 Kaolin-TEG TEG®6s | 87.9 (27.3 to 221.4); median (IQR) | Although R time demonstrates significant strong correlation with rivaroxaban concentration, values within normal range limit clinical utility rendering rivaroxaban concentration the gold standard in measuring anticoagulant effect. |
| Nadtochiy et. al. 2020, U.S.A. ^29^ | Anesth Analg | in vitro | 6 healthy donors | Dabigatran | TEG®5000 RapidTEG^TM^ | 18.75, 37.5, 75, 150, 300, 600, 1200, and 2400 spiked | Dabigatran prolonged R time (significantly > 600 ng/ml) and reduced the dynamics in a dose-dependent manner. |
| Pipilis et. al. 2017, Greece ^34^ | J Thromb Thrombolysis | observational | 75 patients with NVAF | Dabigatran | TEG®5000 CaCl_2_-TEG | 90.4 ± 71.1; mean (± SD) | Plasma dabigatran levels ≥ 200 ng/ml all with R >11 min and level ≤ 30 ng/ml all with R < 8.7 min. Low but significant correlation. |
| Rathburn et. al. 2015, U.S.A. ^36^ | Thromb Res | observational | 22 adults and 10 controls. | Rivaroxaban | TEG®5000 Kaolin-TEG | 118.7 (104.5); mean (SD) | No correlation between serum concentration and R time.  Two patients taking aspirin and another three taking celecoxib. |
| Samama et. al. 2010, France ^37^ | Thromb Haemost | ex vivo | 6 healthy volunteers | Rivaroxaban | TEG®5000 CaCl_2_-TEG | 200, 500, 1000 spiked | A concentration-dependent prolongation of the parameters R and K was observed, without any significant modification of maximum amplitude. |
| Siddiqui et. al. 2019, U.S.A. ^41^ | Clin Appl Thromb Hemost | ex vivo | healthy donors (n = 5 to 7) | Apixaban Betrixaban Edoxaban Rivaroxaban | TEG®5000 CaCl2-TEG | 1000 spiked | DOAC produced effects on TEG parameters. Betrixaban produced the strongest effects and the R time were higher than with other factor Xa inhibitors. Apixaban showed relatively weaker effects than the other factor Xa agents. |
| Solbeck et. al. 2014, Denmark ^45^ | Int J Cardiol | in vitro | 10 healthy donors | Dabigatran | TEG®5000 Kaolin-TEG | 200 spiked | At plasma concentration of 200 ng/ml: Compared to baseline, all parameters were in a hypo coagulable direction. |
| Solbeck et. al. 2016, Denmark ^44^ | Int J Cardiol | in vitro | 8 healthy donors | Dabigatran | TEG®5000 Kaolin-TEG | 0, 50, 100, 200, 400 spiked | A significant overall increase R time was found across increasing dabigatran concentrations. R correlated strongly with plasma concentration. |
| Solbeck et. al. 2018, Denmark ^43^ | Scand J Clin Lab Invest | observational | 35 patients with NVAF | Dabigatran | TEG®5000 Kaolin-TEG | 268.5 (54 to 837), 179.2 (26 to 687); median (IQR) | All patients had prolonged R times. Large individual variation of the anticoagulant response was observed. |
| Spinthakis et. al. 2019, United Kingdom ^46^ | Europace | observational, cross-sectional | 60 patients with NVAF | Apixaban | TEG®5000 Kaolin-TEG | 152.9 (32.9 to 317.9); 125.85 (40.6 to 344.6) median (IQR) | There were no significant relationships between apixaban levels and TEG parameters. |
| Voukalis et. al. 2021, United Kingdom ^53^ | Thromb Res | prospective | 60 patients with AF naive and three months after treatment | Apixaban | TEG®5000 Kaolin-TEG | *NA* | R time (6.8 min) was significantly prolonged. |

**Abbreviations** - Assays: **Kaolin-TEG** intrinsic activated assay; **CaCl_2_ TEG** contains calcium chloride solution**; RapidTEG^TM^** activates and accelerates the clotting process; **AFXa**: anti-factor Xa channel; **DTI**: direct thrombin inhibitor channel. Parameters - **R** reaction time; **Ks** kinetic time; **CL** clot lysis; **CLT** clot lysis time.

**DOAC** direct oral anticoagulant; **R** rivaroxaban; **A** apixaban; **D** dabigatran; **AF** atrial fibrillation; **NVAF** non valvular atrial fibrillation; **NA** not applicable; **IQR** interquartile range.

**Supplementary** **Table 2.3:** Details of included studies for ClotPro®.

| **Author, Year, and Country** | **Journal** | **Study design** | **Population** | **DOAC** | **Assay** | **Plasma concentration of DOAC (ng/mL)** | **Main result** |
| --- | --- | --- | --- | --- | --- | --- | --- |
| Fong et. al. 2020, Malaysia ^14^ | Clin Appl Thromb Hemost | prospective, single-arm, open-label | 118 patients with NVAF | Dabigatran | ECA-test | 59.8 +/- 50.9 | ECA-CT was moderately correlated with trough plasma dabigatran concentration. |
| Groene et.al. 2021, Germany ^16^ | Thrombosis Journal | observational prospective | 70 patients, 10 healthy volunteers / patients with concomitant platelet inhibitors | Apixaban Edoxaban Rivaroxaban Dabigatran | EX-test IN-test RVV-test ECA-test | *NA* | Significant prolongation of CT EX-test and CT IN-test. Plasma concentrations (anti-XA activity) of FXa inhibitors correlated with CT RVV-test. No significance for apixaban. ECA-CT correlated strongly with dabigatran plasma concentration. |
| Oberladstätter et. al. 2020, Austria ^30^ | Anaesthesia | observational prospective | 57 trauma patients on apixaban, 30 on edoxaban, 50 on rivaroxaban, 66 on dabigatran | Apixaban Edoxaban Rivaroxaban Dabigatran | RVV-test ECA-test | A: 0 to 400; E: 0 to 450; R: 0 to 650; D: 0 to 375 approximately | Strong positive correlations between plasma drug levels and CT values. Cut-off values for detecting clinically relevant drug levels showed high levels of sensitivity and specificity. |

**Abbreviations** - Assays: **RVV-test** Russell’s viper venom activated test; **ECA-test** ecarin activated test. **CT** clotting time; **DOAC** direct oral anticoagulant; **R** rivaroxaban; **A** apixaban; **E** edoxaban; **D** dabigatran; **NVAF** non valvular atrial fibrillation; **NA** not applicable.

**References**

1. Adelmann D, Wiegele M, Wohlgemuth RK, et al. Measuring the activity of apixaban and rivaroxaban with rotational thrombelastometry. Article. *Thrombosis research*. 2014;134(4):918-923. doi:10.1016/j.thromres.2014.08.006

2. Aho A, Byrne K. The effect of dabigatran on the kaolin-activated whole blood thromboelastogram. *Anaesthesia and intensive care*. Nov 2016;44(6):729-733. doi:10.1177/0310057x1604400607

3. Aranda VF, Derogis PBM, Sanches LR, et al. Diagnostic accuracy of thromboelastometry and its correlation with the HPLC-MS/MS quantification test. *Brazilian journal of medical and biological research = Revista brasileira de pesquisas medicas e biologicas*. Apr 8 2019;52(4):e8006. doi:10.1590/1414-431x20198006

4. Artang R, Dias JD, Walsh M, et al. Measurement of Anticoagulation in Patients on Dabigatran, Rivaroxaban, and Apixaban Therapy by Novel Automated Thrombelastography. *TH Open*. Oct 2021;5(4):e570-e576. doi:10.1055/a-1692-1415

5. Artang R, Anderson M, Nielsen JD. Fully automated thromboelastograph TEG 6s to measure anticoagulant effects of direct oral anticoagulants in healthy male volunteers. *Research and practice in thrombosis and haemostasis*. Jul 2019;3(3):391-396. doi:10.1002/rth2.12206

6. Bliden KP, Chaudhary R, Mohammed N, et al. Determination of non-Vitamin K oral anticoagulant (NOAC) effects using a new-generation thrombelastography TEG 6s system. *Journal of thrombosis and thrombolysis*. May 2017;43(4):437-445. doi:10.1007/s11239-017-1477-1

7. Casutt M, Konrad C, Schuepfer G. Effect of rivaroxaban on blood coagulation using the viscoelastic coagulation test ROTEM™. *Der Anaesthesist*. Nov 2012;61(11):948-53. doi:10.1007/s00101-012-2091-4

8. Chojnowski K, Górski T, Robak M, Treliński J. Effects of rivaroxaban therapy on ROTEM coagulation parameters in patients with venous thromboembolism. Article. *Advances in Clinical and Experimental Medicine*. 2015;24(6):995-1000. doi:10.17219/acem/42147

9. Comuth WJ, Henriksen L, van de Kerkhof D, et al. Comprehensive characteristics of the anticoagulant activity of dabigatran in relation to its plasma concentration. *Thrombosis research*. Apr 2018;164:32-39. doi:10.1016/j.thromres.2018.02.141

10. Dias JD, Lopez-Espina CG, Ippolito J, et al. Rapid point-of-care detection and classification of direct-acting oral anticoagulants with the TEG 6s: Implications for trauma and acute care surgery. *The journal of trauma and acute care surgery*. Aug 2019;87(2):364-370. doi:10.1097/ta.0000000000002357

11. Dias JD, Norem K, Doorneweerd DD, Thurer RL, Popovsky MA, Omert LA. Use of Thromboelastography (TEG) for Detection of New Oral Anticoagulants. Article. *Archives of pathology & laboratory medicine*. 2015;139(5):665-673. doi:10.5858/arpa.2014-0170-OA

12. Eller T, Busse J, Dittrich M, et al. Dabigatran, rivaroxaban, apixaban, argatroban and fondaparinux and their effects on coagulation POC and platelet function tests. Article. *Clinical Chemistry and Laboratory Medicine*. 2014;52(6):835-844. doi:10.1515/cclm-2013-0936

13. Escolar G, Fernandez-Gallego V, Arellano-Rodrigo E, et al. Reversal of apixaban induced alterations in hemostasis by different coagulation factor concentrates: Significance of studies In Vitro with circulating human blood. Article. *PloS one*. 2013;8(11)e78696. doi:10.1371/journal.pone.0078696

14. Fong AYY, Tiong LL, Tan SSN, et al. Effect of Dabigatran on Clotting Time in the Clotpro Ecarin Clotting Assay: A Prospective, Single-Arm, Open-Label Study. *Clinical and applied thrombosis/hemostasis : official journal of the International Academy of Clinical and Applied Thrombosis/Hemostasis*. Jan-Dec 2020;26:1076029620972473. doi:10.1177/1076029620972473

15. Fontana P, Alberio L, Angelillo-Scherrer A, et al. Impact of rivaroxaban on point-of-care assays. *Thrombosis research*. May 2017;153:65-70. doi:10.1016/j.thromres.2017.03.019

16. Groene P, Wagner D, Kammerer T, et al. Viscoelastometry for detecting oral anticoagulants. *Thrombosis journal*. Mar 16 2021;19(1):18. doi:10.1186/s12959-021-00267-w

17. Havrdová M, Saari TI, Jalonen J, et al. Relationship of Edoxaban Plasma Concentration and Blood Coagulation in Healthy Volunteers Using Standard Laboratory Tests and Viscoelastic Analysis. *Journal of clinical pharmacology*. Oct 7 2020;doi:10.1002/jcph.1758

18. Henskens YMC, Gulpen AJW, van Oerle R, et al. Detecting clinically relevant rivaroxaban or dabigatran levels by routine coagulation tests or thromboelastography in a cohort of patients with atrial fibrillation. *Thrombosis journal*. 2018;16:3. doi:10.1186/s12959-017-0160-2

19. Herrmann R, Thom J, Wood A, Phillips M, Muhammad S, Baker R. Thrombin generation using the calibrated automated thrombinoscope to assess reversibility of dabigatran and rivaroxaban. Article. *Thrombosis and haemostasis*. 2014;111(5):989-995. doi:10.1160/TH13-07-0607

20. Jenrette J, Schwarz K, Trujillo T, Ray L. Evaluation of direct oral anticoagulant use on thromboelastography in an emergency department population. *The American journal of emergency medicine*. Feb 2022;52:191-195. doi:10.1016/j.ajem.2021.12.011

21. Kaaber AB, Jans Ø, Dziegiel MH, Stensballe J, Johansson PI. Managing patients on direct factor Xa inhibitors with rapid thrombelastography. *Scandinavian journal of clinical and laboratory investigation*. Dec 2021;81(8):661-669. doi:10.1080/00365513.2021.2003855

22. Klages M, Raimann FJ, Philipp AL, Lindhoff-Last E, Zacharowski K, Mutlak H. Direct oral anticoagulants in point-of-care monitoring: an ex-vivo study. *Minerva anestesiologica*. May 2021;87(5):514-522. doi:10.23736/s0375-9393.21.14788-1

23. Kopytek M, Zabczyk M, Natorska J, Malinowski KP, Undas A. Effects of direct oral anticoagulants on thromboelastographic parameters and fibrin clot properties in patients with venous thromboembolism. *Journal of physiology and pharmacology : an official journal of the Polish Physiological Society*. Feb 2020;71(1)doi:10.26402/jpp.2020.1.03

24. Körber MK, Langer E, Köhr M, Wernecke KD, Korte W, Von Heymann C. In vitro and ex vivo Measurement of Prophylactic Dabigatran Concentrations with a New Ecarin-Based Thromboelastometry Test. *Transfusion Medicine and Hemotherapy*. 2017;44(2):100-105. doi:10.1159/000470622

25. Körber MK, Langer E, Ziemer S, Perzborn E, Gericke C, Von Heymann C. Measurement and reversal of prophylactic and therapeutic peak levels of rivaroxaban: An in vitro study. Conference Paper. *Clinical and Applied Thrombosis/Hemostasis*. 2014;20(7):735-740. doi:10.1177/1076029613494468

26. Kyriakou E, Katogiannis K, Ikonomidis I, et al. Laboratory Assessment of the Anticoagulant Activity of Apixaban in Patients With Nonvalvular Atrial Fibrillation. Article. *Clinical and Applied Thrombosis/Hemostasis*. 2018;24(9_suppl):194S-201S. doi:10.1177/1076029618802364

27. Kyriakou E, Ikonomidis I, Stylos D, et al. Laboratory Assessment of the Anticoagulant Activity of Dabigatran. Article. *Clinical and Applied Thrombosis/Hemostasis*. 2015;21(5):434-445. doi:10.1177/1076029614564209

28. Myers SP, Dyer MR, Hassoune A, et al. Correlation of Thromboelastography with Apparent Rivaroxaban Concentration: Has Point-of-Care Testing Improved? *Anesthesiology*. Feb 2020;132(2):280-290. doi:10.1097/aln.0000000000003061

29. Nadtochiy SM, Baldzizhar A, Stefanos T, et al. High-Dose Dabigatran Is an Effective Anticoagulant for Simulated Cardiopulmonary Bypass Using Human Blood. *Anesthesia and analgesia*. Aug 20 2020;doi:10.1213/ane.0000000000005089

30. Oberladstätter D, Voelckel W, Schlimp C, et al. A prospective observational study of the rapid detection of clinically-relevant plasma direct oral anticoagulant levels following acute traumatic injury. *Anaesthesia*. Sep 18 2020;doi:10.1111/anae.15254

31. Oswald E, Velik-Salchner C, Innerhofer P, et al. Results of rotational thromboelastometry, coagulation activation markers and thrombin generation assays in orthopedic patients during thromboprophylaxis with rivaroxaban and enoxaparin: A prospective cohort study. Article. *Blood Coagulation and Fibrinolysis*. 2015;26(2):136-144. doi:10.1097/MBC.0000000000000203

32. Pailleret C, Jourdi G, Siguret V, et al. Modified ROTEM for the detection of rivaroxaban and apixaban anticoagulant activity in whole blood: A diagnostic test study. *European journal of anaesthesiology*. Jun 2019;36(6):449-456. doi:10.1097/eja.0000000000000903

33. Perzborn E, Heitmeier S, Laux V, Buchmüller A. Reversal of rivaroxaban-induced anticoagulation with prothrombin complex concentrate, activated prothrombin complex concentrate and recombinant activated factor VII in vitro. *Thrombosis research*. Apr 2014;133(4):671-81. doi:10.1016/j.thromres.2014.01.017

34. Pipilis A, Makrygiannis S, Anagnostou G, et al. Dabigatran plasma levels, aPTT and thromboelastography in patients with AF: implications for allowing early non-elective surgical procedures. *Journal of thrombosis and thrombolysis*. Jul 2017;44(1):9-13. doi:10.1007/s11239-017-1503-3

35. Pujadas-Mestres L, Lopez-Vilchez I, Arellano-Rodrigo E, et al. Differential inhibitory action of apixaban on platelet and fibrin components of forming thrombi: Studies with circulating blood and in a platelet-based model of thrombin generation. *PloS one*. 2017;12(2):e0171486. doi:10.1371/journal.pone.0171486

36. Rathbun S, Tafur A, Grant R, Esmon N, Mauer K, Marlar RA. Comparison of methods to determine rivaroxaban anti-factor Xa activity. Article. *Thrombosis research*. 2015;135(2):394-397. doi:10.1016/j.thromres.2014.11.017

37. Samama MM, Martinoli JL, LeFlem L, et al. Assessment of laboratory assays to measure rivaroxaban - An oral, direct factor Xa inhibitor. Article. *Thrombosis and haemostasis*. 2010;103(4):815-825. doi:10.1160/TH09-03-0176

38. Schenk B, Würtinger P, Streif W, Sturm W, Fries D, Bachler M. Ex vivo reversal of effects of rivaroxaban evaluated using thromboelastometry and thrombin generation assay. *British journal of anaesthesia*. Nov 2016;117(5):583-591. doi:10.1093/bja/aew259

39. Schmidt K, Krüger K, Langer E, et al. Reversal of apixaban induced alterations in haemostasis by different coagulation factor concentrates in patients after hip or knee replacement surgery. *Blood transfusion = Trasfusione del sangue*. Mar 2019;17(2):157-162. doi:10.2450/2018.0028-18

40. Seyve L, Richarme C, Polack B, Marlu R. Impact of four direct oral anticoagulants on rotational thromboelastometry (ROTEM). *International journal of laboratory hematology*. Feb 2018;40(1):84-93. doi:10.1111/ijlh.12744

41. Siddiqui F, Hoppensteadt D, Jeske W, Iqbal O, Tafur A, Fareed J. Factor Xa Inhibitory Profile of Apixaban, Betrixaban, Edoxaban, and Rivaroxaban Does Not Fully Reflect Their Biologic Spectrum. *Clinical and applied thrombosis/hemostasis : official journal of the International Academy of Clinical and Applied Thrombosis/Hemostasis*. Jan-Dec 2019;25:1076029619847524. doi:10.1177/1076029619847524

42. Sokol J, Nehaj F, Ivankova J, et al. Impact of Dabigatran Treatment on Rotation Thromboelastometry. *Clinical and applied thrombosis/hemostasis : official journal of the International Academy of Clinical and Applied Thrombosis/Hemostasis*. Jan-Dec 2021;27:1076029620983902. doi:10.1177/1076029620983902

43. Solbeck S, Jensen AS, Maschmann C, Stensballe J, Ostrowski SR, Johansson PI. The anticoagulant effect of therapeutic levels of dabigatran in atrial fibrillation evaluated by thrombelastography (TEG(®)), Hemoclot Thrombin Inhibitor (HTI) assay and Ecarin Clotting Time (ECT). *Scandinavian journal of clinical and laboratory investigation*. Feb-Apr 2018;78(1-2):25-30. doi:10.1080/00365513.2017.1408138

44. Solbeck S, Ostrowski SR, Stensballe J, Johansson PI. Thrombelastography detects dabigatran at therapeutic concentrations in vitro to the same extent as gold-standard tests. Article. *International journal of cardiology*. 2016;208:14-18. doi:10.1016/j.ijcard.2016.01.148

45. Solbeck S, Meyer MA, Johansson PI, et al. Monitoring of dabigatran anticoagulation and its reversal in vitro by thrombelastography. *International journal of cardiology*. Oct 20 2014;176(3):794-9. doi:10.1016/j.ijcard.2014.07.084

46. Spinthakis N, Gue Y, Farag M, et al. Apixaban enhances endogenous fibrinolysis in patients with atrial fibrillation. *Europace : European pacing, arrhythmias, and cardiac electrophysiology : journal of the working groups on cardiac pacing, arrhythmias, and cardiac cellular electrophysiology of the European Society of Cardiology*. Sep 1 2019;21(9):1297-1306. doi:10.1093/europace/euz176

47. Takeshita S, Tanaka KA, Sawa T, Sanda M, Mizobe T, Ogawa S. Whole Blood Point-of-Care Testing for Incomplete Reversal With Idarucizumab in Supratherapeutic Dabigatran. *Anesthesia and analgesia*. Feb 2020;130(2):535-541. doi:10.1213/ane.0000000000004419

48. Taune V, Skeppholm M, Ågren A, et al. Rapid determination of anticoagulating effects of dabigatran in whole blood with rotational thromboelastometry and a thrombin-based trigger. *Journal of thrombosis and haemostasis : JTH*. Dec 2018;16(12):2462-2470. doi:10.1111/jth.14308

49. Taune V, Wallén H, Ågren A, et al. Whole blood coagulation assays ROTEM and T-TAS to monitor dabigatran treatment. *Thrombosis research*. May 2017;153:76-82. doi:10.1016/j.thromres.2017.03.018

50. Tsantes AE, Kyriakou E, Bonovas S, et al. Impact of dabigatran on platelet function and fibrinolysis. Article. *Journal of the Neurological Sciences*. 2015;357(1-2):204-208. doi:10.1016/j.jns.2015.07.031

51. Tsantes AE, Kyriakou E, Ikonomidis I, et al. Comparative Assessment of the Anticoagulant Activity of Rivaroxaban and Dabigatran in Patients With Nonvalvular Atrial Fibrillation: A Noninterventional Study. *Medicine*. Apr 2016;95(14):e3037. doi:10.1097/md.0000000000003037

52. Vedovati MC, Mosconi MG, Isidori F, Agnelli G, Becattini C. Global thromboelastometry in patients receiving direct oral anticoagulants: the RO-DOA study. *Journal of thrombosis and thrombolysis*. Feb 2020;49(2):251-258. doi:10.1007/s11239-019-01956-0

53. Voukalis C, Lip GYH, Shantsila E. Effects of antithrombotic drugs on the prothrombotic state in patients with atrial fibrillation: The west Birmingham atrial fibrillation project. *Thrombosis research*. Apr 2021;200:149-155. doi:10.1016/j.thromres.2021.02.005
